# Multichannel Detrended Fluctuation Analysis Reveals Synchronized Patterns of Spontaneous Spinal Activity in Anesthetized Cats

**DOI:** 10.1371/journal.pone.0026449

**Published:** 2011-10-27

**Authors:** Erika E. Rodríguez, Enrique Hernández-Lemus, Benjamín A. Itzá-Ortiz, Ismael Jiménez, Pablo Rudomín

**Affiliations:** 1 Department of Physiology, Biophysics and Neurosciences, Center for Research and Advanced Studies (CINVESTAV), Mexico City, Mexico; 2 Center for Research in Mathematics, Autonomous University of Hidalgo (UAEH), Pachuca, Mexico; 3 Computational Genomics Department, National Institute of Genomic Medicine, Mexico City, Mexico; 4 Center for Complexity Sciences, National Autonomous University of Mexico (UNAM), Mexico City, Mexico; Universidad Veracruzana, Mexico

## Abstract

The analysis of the interaction and synchronization of relatively large ensembles of neurons is fundamental for the understanding of complex functions of the nervous system. It is known that the temporal synchronization of neural ensembles is involved in the generation of specific motor, sensory or cognitive processes. Also, the intersegmental coherence of spinal spontaneous activity may indicate the existence of synaptic neural pathways between different pairs of lumbar segments. In this study we present a multichannel version of the detrended fluctuation analysis method (mDFA) to analyze the correlation dynamics of spontaneous spinal activity (SSA) from time series analysis. This method together with the classical detrended fluctuation analysis (DFA) were used to find out whether the SSA recorded in one or several segments in the spinal cord of the anesthetized cat occurs either in a random or in an organized manner. Our results are consistent with a non-random organization of the sets of neurons involved in the generation of spontaneous cord dorsum potentials (CDPs) recorded either from one lumbar segment (DFA-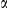 mean = 1.04
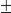
0.09) or simultaneously from several lumbar segments (mDFA-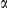 mean = 1.01
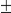
0.06), where 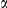 = 0.5 indicates randomness while 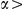
0.5 indicates long-term correlations. To test the sensitivity of the mDFA method we also examined the effects of small spinal lesions aimed to partially interrupt connectivity between neighboring lumbosacral segments. We found that the synchronization and correlation between the CDPs recorded from the L5 and L6 segments in both sides of the spinal cord were reduced when a lesion comprising the left dorsal quadrant was performed between the segments L5 and L6 (mDFA-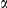 = 0.992 as compared to initial conditions mDFA-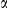 = 1.186). The synchronization and correlation were reduced even further after a similar additional right spinal lesion (mDFA-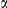 = 0.924). In contrast to the classical methods, such as correlation and coherence quantification that define a relation between two sets of data, the mDFA method properly reveals the synchronization of multiple groups of neurons in several segments of the spinal cord. This method is envisaged as a useful tool to characterize the structure of higher order ensembles of cord dorsum spontaneous potentials after spinal cord or peripheral nerve lesions.

## Introduction

It has been claimed that the central nervous system is an intrinsically synchronized dynamical system [1]. In this sense, synchronization refers to functional coordination characterized by rhythmic or repetitive neural activity. In the case of neural ensembles, synchronized activity of multiple neurons can give rise to macroscopic oscillations, which can be experimentally observed by means of both electrophysiological records and imaging techniques [2]. Synchronicity in the neuronal activity appears to be an important process for information transmission. It allows the coordination of the activity of single neurons with either part or the entire population of neurons involved in the generation of specific motor, sensory or cognitive processes. Synchronization phenomena have been analyzed during different embryonic stages of animals and it is considered as a probable training or preparatory step for neural network control of several physiological processes [3]. For example, developing networks in the chicken become spontaneously active and synchronized during early stages of development and remain this way until hatching. The spontaneous and synchronized activity has been shown to play a fundamental role in the development of neurons and muscles [4]. In the field of cognitive neuroscience, reports have been given of enhanced synchronization in groups and individual neurons during the attentive and expectation stages for visual stimuli [1]. Synchronicity of multiple regions has been found in the visual and parietal cortices of cats performing go and no go tasks [5]. These tasks show a strong dependency in the low frequency range for 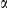 and 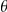 signals (4–12 Hz) in electroencephalogram recordings. This behavior is stronger during go tasks than under no go tasks and such observations have been related to expectancy [6], [7]. In human subjects under situations involving object [8], learning [9], language processing [10] and emotional evaluation [11], an enhancement in high-frequency 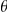-components have been observed. This has been taken as evidence that synchronization phenomena are the result of intrinsic dynamic interaction of sensory circuits [1].

Spontaneous cord dorsum potentials (CDPs) were first recorded in the spinal cord of the cat more than 60 years ago by Bremer [12] and ten Cate [13]. CDPs are characterized by a noise-like background activity or *basal activity* generated in absence of any stimulation. Mark and Gasteiger reported that spontaneous CDPs persisted after deafferentation or spinalization [14] and suggested they were generated by intrinsic spinal mechanisms. Studies using intact and free moving cats indicated that the spontaneous CDPs were similar to those recorded in anesthetized animals but with lower frequency and smaller amplitude [15].

Manjarrez and colleagues [16] reported the occurrence of spontaneous CDPs in the anesthetized cat that appear simultaneously in a variable number of segments along the spinal cord. They suggested that these potentials were generated by the synchronized activation of dorsal horn neuronal aggregates distributed along the lumbosacral spinal segments [17]. Later on, García et al [18] showed that the synchronization between the spontaneous CDPs recorded from neighboring segments was reduced by an interposed spinal lesion. Studies in humans provide interesting, but mostly descriptive information, on the alterations of the spontaneous CDPs under normal and pathological conditions such as spinal cord and/or nerve lesions [19], [20].

The characterization of a possible structure of spontaneous CDPs simultaneously recorded from several spinal segments, could give some light on the mechanisms involved in their generation and propagation. In this sense it would be very useful to have an analytical method for the analysis of synchronicity in spontaneous activated groups of neurons. In contrast to the classical methods, such as correlation and coherence that define a relation between two sets of data, and the classical detrended fluctuation analysis (DFA) method that examines the correlation dynamics in a single channel, we present a method that is able to characterize the structure of higher order ensembles of spontaneous CDPs in a quantitative manner. Here we present a couple of examples aimed to explore the sensitivity of the mDFA method to characterize changes produced by spinal lesions which partially interrupted connectivity between neighboring lumbosacral segments.

## Analysis

### Detrended Fluctuation Analysis

It is known that a bounded time series could be mapped to a self-similar process by integration. Peng, et al [21] introduced a modified root mean square analysis of the underlying random walk that has been termed detrended fluctuation analysis (DFA). DFA detects self-similar patterns even if they are embedded in an apparent non-stationary frame, and also avoids the spurious detection of artificial self-similarity due to trending of the probability distribution function. The methodology of the DFA is as follows. Start with a time series 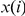 with discrete steps 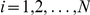. Next, consider the integrated values of this time series, that is,
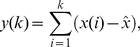
(1)where 
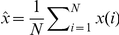
 is the average value of 
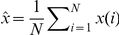
. This integration step maps a time series to a self-similar process. The next goal is now to measure the vertical characteristic scale for the integrated time series, and this is achieved by dividing the integrated time series into boxes of equal length 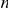. For each one of these boxes a least squares linear fitting of the data (called the *local trend* on that box) is performed. The value of the 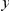 coordinate of the straight line is denoted by 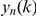. More generally, 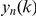 could represent the 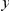 coordinate of a degree 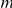 polynomial fitting. This is of particular importance if one wishes to remove not only constant trends or linear terms but also higher order trends. In such a case we refer to the resulting method as DFAm, with 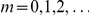, representing the degree of the associated polynomial. In order to *detrend* the integrated time series 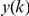, for each box, we subtract the linear local trend 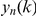. For a given box of size 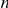, the characteristic length-scale for the fluctuations in the integrated and detrended series is:
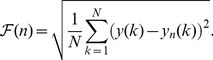
(2)


By plotting the values of 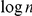 against the values of 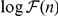, a linear relationship is observed which indicates the presence of a power law, that is to say
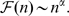
(3)


Hence, the scaling exponent 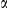 represents the scaling of the fluctuations (recall that in the case of DFAm, a comparison of the different exponents 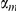 gives information about the order of the trend of the data) and can be approximated as the slope of the line relating 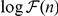 and 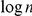. The exponent 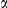 has been called the Hurst exponent and it is used as a measure of long-ranged correlations in time series. The Hurst exponent relates the self-correlations of the time series with the rate at which this decrease *in time* (i.e. as we move along the time series) [22]. DFA has revealed the extent of long-range correlations in apparently irregular time series [21], [23]. A value of 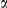 greater than 0.5 indicates the presence of persistent long-range power law correlations. The case 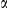 = 1.0 has raised a lot of of interest both from physicists and biologists for many years, it corresponds to 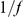 noise [24]. Finally, the case 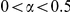 denotes the presence of power-law anti-correlations, such that large values are more likely to be followed by small values and vice versa [21]. DFA is thus a good-standing methodology to detect scaling behavior in observational time series that may be affected by nonstationarities as is the case with neurophysiological signals.

### Multichannel Detrended Fluctuation Analysis

Classical DFA [21], [24] involves time series which are sequences of observations consisting of just one input. The goal of DFA is to assess the long range correlation of the data involved, for example, in the non-stationary heartbeat time series [23] and nonlinear analysis of anesthesia dynamics [25]. Since, the central nervous system is an intrinsically synchronized dynamic system and synchronization of multiple neuronal areas are required to process information, it seems evident the need for a similar analysis that involves time series of several inputs. That is, to assess the long range correlation of multichannel data.

Attempts to apply DFA to multichannel time series have been used in the past to seismic fluctuations data [26] and was also used for multifractal analysis of DNA walks and trails [27]. The role of multidimensionality has also been discussed in relation to multifractality [28]. In the case of reference [27], DFA techniques have been compared with wavelet transform techniques and have been based in the consideration of a d-dimensional random walk. Both reference [26] and reference [27] applied DFA to two dimensional time series and suggested that is possible to describe a formula for multidimensional time series data using their formulas. Kantelhardt, et al [28] also considered the relation between the Hurst exponents and the singularity spectrum via Legendre transformation, the role of statistical deviations in DFA for short time series and the presence of correlated randomness.

Here we present a generalization of DFA that begins with a time series 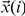 with discrete steps 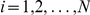, so that each 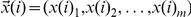 is a 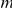-dimensional vector, where 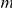 is the number of inputs of each recording. The multichannel DFA (mDFA) could now be implemented by considering the integrated values of this time series as in equation (1), that is,
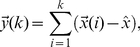
(4)where 
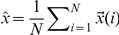
 is the vector consisting of the average values of the components of the vectors 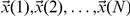 conforming our time series. As consequence of this integration step, we now have a componentwise self-similar process. Next, we measure the vertical characteristic scale for the integrated time series, component by component. To achieve this, we divide each component of the integrated time series into boxes of equal length 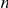. For each one of this boxes and for the data of each component, a least squares linear fitting (called the local trend of the component on that box) is performed. The vector of values of the 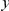 coordinates of the straight lines is denoted by 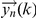. To detrend the integrated time series 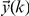, we detrend component by component, so that we subtract 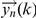. By modifying equation (2) in such a way that individual contributions for the detrended fluctuations of every vector component 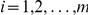 is taken into account, we define
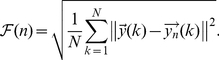
(5)


By plotting the the values of 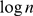 against the values of 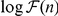, a linear relationship is observed which indicates the presence of a power law, that is to say
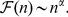
(6)


Hence, the scaling exponent 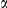 represents the fluctuations and can be approximated as the slope of the line relating 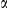 and 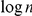, as in the classic DFA situation. A value of 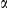 greater than 0.5 indicates the presence of persistent long-range power law correlations, while the case 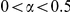 denotes the presence of power-law anti-correlations such that large values are more likely to be followed by small values and vice versa.

## Materials and Methods

### General procedures

The data describe here were obtained from 10 experiments performed in adult cats. Guidelines contained in Principles of Laboratory Animal Care (NIH publications 85-23, revised in 1985) were followed in all cases and the experiments were also approved by the Institutional Bioethical Committee (Protocol number: 0126-03). The animals were initially anesthetized with pentobarbitone sodium (40 mg/kg i.p) and additional doses were given intravenously to maintain an adequate level of anesthesia, tested by assessing that withdrawal reflexes were absent, that the pupils were constricted and the arterial blood pressure was between 100 and 120 mm/Hg. The carotid artery, radial vein, trachea and urinary bladder were cannulated. A solution of 100 mM of sodium bicarbonate with glucose 5% was given i.v. (0.03 ml/min) to prevent acidosis [29]. When necessary, dextran 10% or ethylephrine (Effortil, Boering-Ingelheim) was administered to keep blood pressure above 100 mm/Hg.

The lumbosacral and low thoracic spinal segments were exposed. After the surgical procedures, the animals were transferred to a stereotaxic metal frame allowing immobilization of the spinal cord, paralyzed with pancuronium bromide (0.1 mg/kg) and artificially ventilated. The tidal volume was adjusted to maintain 4% of 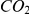 concentration in the exhaled air. To prevent desiccation of the exposed tissues, pools were made with the skin flaps, filled with paraffin oil and maintained between 36 and 37
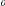
C by means of radiant heat. Usually six ball electrodes were placed on the cord dorsum of the lumbosacral enlargement at different spinal segments to record the spontaneous CDPs against an indifferent electrode placed on the paravertebral muscles (band pass filters 0.3 Hz to 10 kHz). Left and right spinal lesions were made between segments L5 and L6 with particular care to avoid touching the recording electrodes.

### Computational and statistical analysis

Calculations involved in computing DFA scaling exponent were implemented using computational methods included in the PhysioToolkit library [30] via the DFA package and the original DFA. A modified version, termed multichannel detrended fluctuation analysis (mDFA) has been implemented in RapidMiner ver.5.0 according to equations 4, 5 and 6. Source code for the programs may be provided if requested. Recordings of spinal lumbar segments lasted 10 minutes or 1 million points. Statistical Student t-test was used to compare control and lesioned animals. The coherence was calculated using mscohere of MatLab ver. 7.4.

## Results

### Intact neuroaxis and randomized spontaneous CDPs recordings

**Figure 1:**
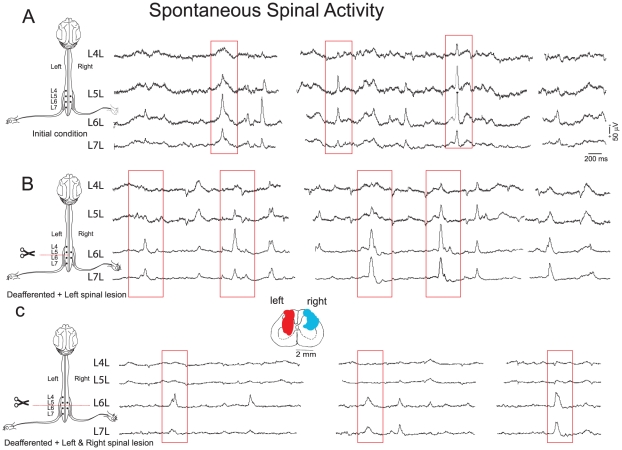
Spontaneous spinal activity with intact and lesioned spinal cord recorded in different spinal segments from the anesthetized cat. **A.** Recordings of the initial conditions which showed synchronization and high correlation in different lumbar segments of CDPs. These are repeated throughout the recording at different time intervals as shown in traces. **B.** Spontaneous CDP recording made 75 minutes after deafferentation and a left spinal lesion between segments L5 and L6 at left side. It is showed a reduction in the synchronization of spontaneous CDPs. **C.** Right spinal lesion made 40 minutes after the left spinal lesion. The synchronization was almost abolished after the second spinal lesion between L5 and L6 at left side. See text for details.

Simultaneous recordings from several segments in the cord dorsum of the lumbosacral enlargement revealed synchronized spontaneous potentials of different shapes and amplitudes. Figure 1A shows the spontaneous CDPs simultaneously recorded from four spinal segments (L4 to L7) in the left side in a preparation with intact neuroaxis. It may be seen that at one time the CDPs recorded in the L5 and L6 segments on the left side were larger than those recorded in the other segments. Later on, simultaneous CDPs appeared in all segments. Particular combinations of spontaneous CDPs could be observed several times during a single recording period (see also Manjarrez, et. al. [16]). Since the CDPs last approximately from 100 ms to 300 ms, we used windows of this size in order to include the low frequency components of these potentials.

**Figure 2:**
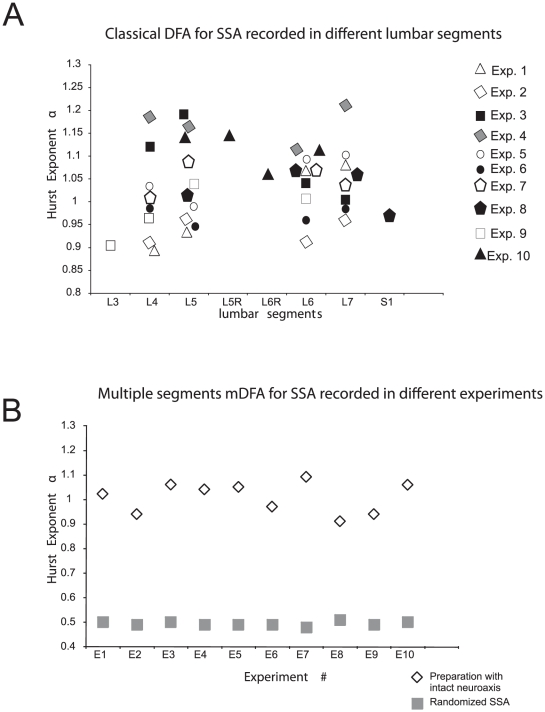
Detrended Fluctuation Analysis. **A.** Classical DFA computed for spontaneous spinal activity (SSA). In 10 experiments spontaneous CDPs (Cord Dorsum Potentials) recorded from a single spinal segment under initial conditions showed a Hurst exponent value of DFA-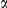 = 1.04
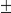
0.09 (mean 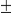 sd). The recordings of single segments were among L3 through S1 depending on the experiments protocol. The R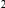 line averaged was 0.98. See text for details. **B.** The Hurst exponent values (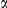
's) were obtained from mDFA for multiple spinal segments for four lumbar segments in 10 experiments under initial conditions (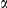 = 1.01
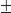
0.06) with a R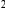 line = 0.98. Following randomization, the same set of data showed mDFA-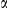 = 0.49
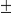
0.01, with an average R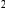 for line-fitting higher than 0.99.

In 10 preparations with intact neuroaxis we registered the spontaneous activity simultaneously from several segments (from L3 to S1) and for each single spinal segment we calculated the Hurst exponent with the classical DFA (Figure 2A). The mean value of all Hurst exponents was 1.04
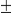
0.09 (mean 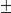 sd). In addition, we calculated the Hurst exponent by using the method mDFA, for signals recorded simultaneously from 4 spinal segments. We obtained a mean value of 1.01
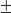
0.06.

According to the criteria stated in Sections detrended fluctuation analysis and multichannel detrended fluctuation analysis, where 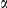 = 0.5 indicates randomness while 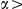
0.5 indicates long-term correlations, the classical DFA data depicted in Figure 2A as well as the mDFA data of Figure 2B indicate that the spontaneous CDPs had a non-random structure suggesting that they arise from a structured neuronal organization. In contrast, when randomized, the same set of data showed mean mDFA-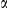 = 0.49
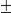
0.01 (Figure 2B), while the mean of DFA-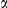 = 0.49
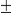
0.02.

### Decoupling produced by spinal cord lesions

Spontaneous CDPs were simultaneously recorded from several spinal segments before and after a first lesion in the left side between segments L5 and L6 and a second lesion in the right side, also between segments L5 and L6, both comprising the dorsolateral quadrant of the spinal cord. In order to exclude possible influences from peripheral inputs, these recordings were taken after the bilateral transection of the L3-S1 dorsal roots. After the left spinal lesion, the CDPs recorded from the four segments still remain synchronized, but to a lesser degree than before the lesion (Figure 1B). After the additional lesion of the right dorsal quadrant, the synchronization between these segments was significantly reduced (Figure 1C). Yet, there was still a significant synchronicity between potentials recorded from pairs of segments either rostrally or caudally to the lesion. (i.e. L4 and L5 or L6 and L7). This could be interpreted as evidence for the hypothesis that left and right groups of neurons in different segments are both interconnected by pathways running in the same as well as in the opposite sides (see García et al [18] ).

#### Changes in the fractal structure of spontaneous CDPs with an interposed spinal lesion

**Figure 3:**
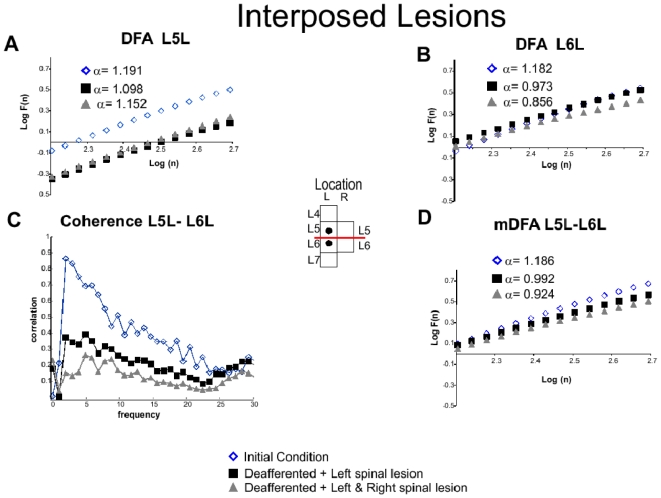
Recordings of spontaneous spinal activity with an interposed lesion in initial conditions, deafferented and spinal lesions. **A.** Classical DFA for L5L. **Insert:** Location of electrodes. **B.** Classical DFA for L6L. **C.** Coherence between L5L–L6L. **D.** mDFA L5L–L6L. The coherence was reduced after lesions as well as mDFA Hurst exponents. The regression line fit, 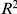 was above 0.99 in all cases. The spinal lesion reduced the fluctuations significantly. More details in text.

The DFA of potentials recorded in the L5L and L6L segments were reduced after the first spinal lesion in the same side, possibly because of interruption of interconnecting pathways (Figure 3A and B, black squares). On the other hand, the right spinal lesion had opposite effects on the Hurst values of the potentials recorded rostrally and caudally to the lesion. Namely, the Hurst values of the L5L were increased from 1.098 to 1.152 and those of the L6L were reduced from 0.973 to 0.856 (Figure 3A and B, triangles). It is to be noted that these spinal lesions had different effects on 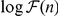, which is an index of the fluctuations of the potentials, which varied according to the window size. In the case of the potentials recorded from L5L, both spinal lesions produced a marked reduction of 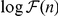. In contrast, for L6L 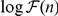 remained essentially the same. The Hurst values calculated with the mDFA for the L5L–L6L ensemble were also reduced after the spinal lesions when compared with the values obtained in the preparation with intact neuroaxis (Figure 3D and Table 1). 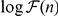, was also reduced, but to a smaller, yet statistically significant extent, particularly for the largest window sizes. For comparison, we also examined the changes in coherence between the L5L and L6L potentials produced by the spinal lesions. (Coherence is defined as the correlation of the frequency components). We found that after each of the two spinal lesions the coherence was reduced throughout the whole frequency range (figure 3C). Most likely, the remaining correlation was introduced by the pathways spared by the spinal lesions. These changes of the fractal correlation structure are believed to have a functional origin since it has been proposed that the segmental synchronizations of dorsal root reflexes and dorsal root potentials are mediated by the spinal cord connections through the lateral funiculi in rats and hamsters [31], [32]. Therefore, if the dorsolateral fasciculus (DLF) mediates the intersegmental synchronization in the spinal cord of the anesthetized cat then a lesion comprising DLF would decouple the adjacent segments where the lesion was made. Partial spinal lesion reduced the amplitude of spontaneous CDPs for the segments with an interposed lesion (Figure 1B). Total spinal lesion almost abolished the temporal synchronization between the adjacent segments where the lesion was located. The occurrence of spontaneous CDPs in the segments L5 and L6 was significantly reduced by the complete spinal lesion (Figure 1C). On the other hand, coherence showed a significantly reduced correlation in the low frequency components which are related to the spontaneous CDPs. Furthermore, the mDFA shows a reduction when compared to the intact neuroaxis and fluctuations were significantly reduced for the windows with sizes that comprised the spontaneous CDPs (Figure 3). Since the reduction in the correlation of spontaneous CDPs between the segments with an interposed lesion (Figure 1C) reveals a reduction of mDFA Hurst values (Figure 3D), this points out that spinal cord lesions changed the fractal structure of spontaneous cord dorsum potentials.

**Table 1:** 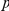
-values for significance tests of fluctuations reduction caused by lesions in L5L and L6L.

Interposed lesions	Left spinal lesion	Right spinal lesion
DFA L5L	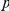	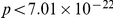
DFA L6L	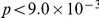	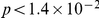
mDFA L5L-L6L	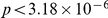	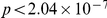

#### Changes in the fractal structure of spontaneous CDPs recorded rostral to lesions

**Figure 4:**
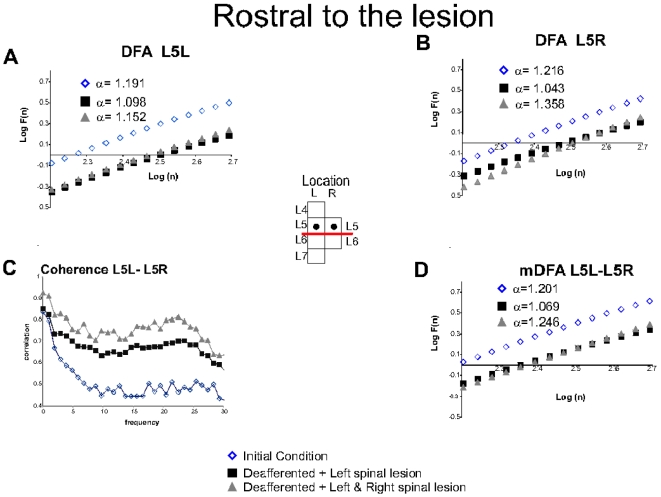
Recordings of spontaneous spinal activity above the lesion with initial conditions, deafferented and spinal lesions. **A.** Classical DFA for L5L. **Insert:** Boxes indicating the location of electrodes L5 left and L5 right (black dots). **B.** Classical DFA for L5R. **C.** Coherence for L5L- L5R increased after lesions. **D.** mDFA L5L-L5R. The regression line fit, 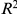 was higher than 0.99 in all cases. Note that the spinal lesion significantly reduced the logarithm of the fluctuations of signals (P
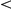
0.001). The fractal structure increased after the second lesion for both classical DFA and mDFA for the recordings above the lesion. See details in text.

The DFA of potentials recorded in L5L and L5R segments, both above the lesion, were reduced after the first left spinal lesion (Figure 4A and B, black squares) and slightly increased after the second lesion. More specifically, the Hurst values of the L5L and L5R were increased from 1.098 to 1.152 and from 1.043 to 1.358, respectively (Figure 4A and B, triangles). In addition, 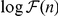 was reduced throughout the whole window range (Table 2). The Hurst values calculated with the mDFA for the L5L - L5R ensemble were also reduced after the first left spinal lesion when compared with values obtained while the neuroaxis was still intact (from 1.201 to 1.069) and subsequently increased after the second spinal lesion to 1.246 (Figure 4D and Table 2). 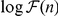 was also significantly reduced, throughout the whole window range (Table 2). Quite interestingly, in this case the coherence between the potentials recorded from L5L and L5R was increased throughout the whole frequency range (Figure 4C).

**Table 2:** 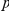
-values for significance tests of fluctuations reduction caused by lesions in L5L and L5R.

Rostral to the lesion	Left spinal lesion	Right spinal lesion
DFA L5L	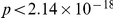	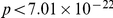
DFA L5R	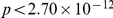	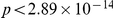
mDFA L5L-L5R	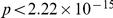	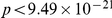

#### Changes in the fractal structure of spontaneous CDPs recorded caudal to the lesion

**Figure 5:**
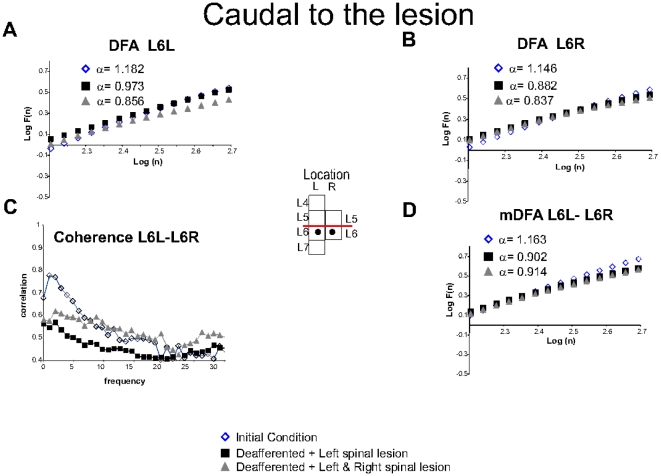
Spontaneous spinal recordings located below the lesion. **A.** classical DFA for L6L. **Insert:** Location of electrodes. **B.** Classical DFA for L6R. **C.** Coherence between L6L-L6R was reduced for first lesion and followed by an increased for the second lesion as well as mDFA. **D.** mDFA for L6L and L6R. The regression line fit, 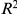 was above 0.99 in all cases. More details in text. Note that the spinal lesion reduced the fluctuations, but such effect was not significant.

The DFA of potentials recorded in L6L and L6R were reduced after each of the two spinal lesions. Namely, the Hurst values of the L6L were reduced from 1.182 to 0.973 and 0.856 and those of the L6R from 1.146 to 0.882 and 0.837 (Figure 5A and B, squares and triangles). However, 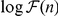 for both L6L and L6R remained essentially the same (Table 3). A similar behavior was seen with the Hurst values calculated with the mDFA for the L6L-L6R ensemble (from 1.163 to 0.902 and 0.914 after the first and second lesions, respectively (Figure 5D and Table 3). 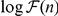, was not significantly changed. In this case, the coherence between the L6L and L6R potentials was reduced after the first spinal lesion and increased after the second lesion as the mDFA of L6L-L6R.(Figure 5C and D).

**Table 3:** 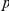
-values for significance tests of fluctuations reduction caused by lesions in L6L and L6R.

Caudal to the lesion	Left spinal lesion	Right spinal lesion
DFA L6L	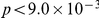	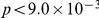
DFA L6R	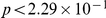	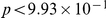
mDFA L6L-L6R	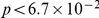	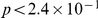

#### mDFA for multiple lumbar segments recordings

**Figure 6:**
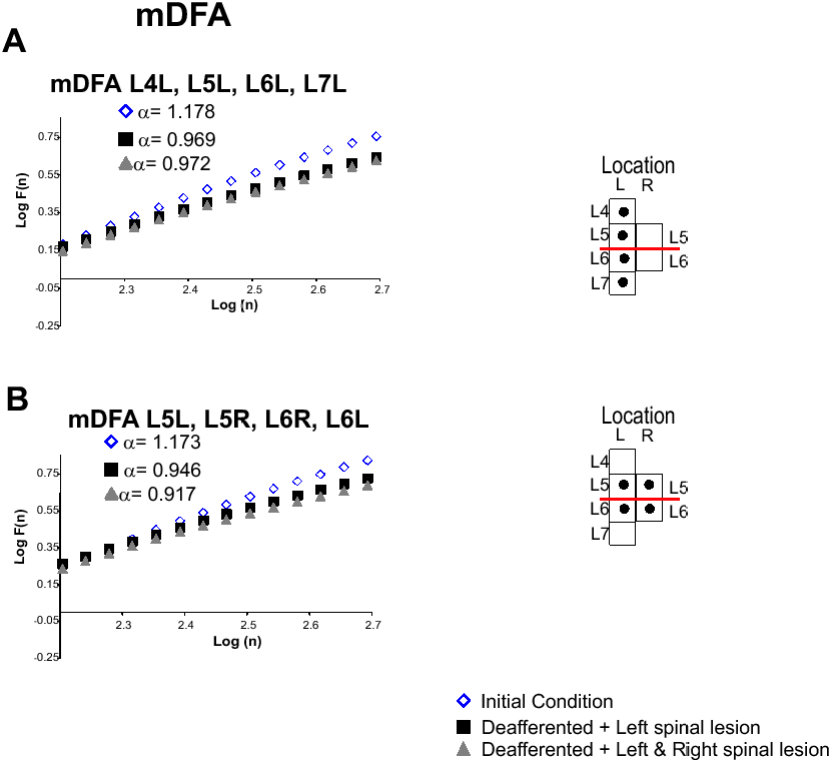
mDFA analysis of multiple recordings of the spontaneous activity in the cat spinal cord. **A.** mDFA for L4 through L7 located in the left size. **Inserts:** Location of electrodes. **B.** mDFA for CDPs recordings near to the lesion, L5L-L5R-L6R-L6L. The regression line fit, 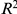 was above 0.99 in all cases. More details in text. The spinal lesion reduced significantly the fluctuations.

One of the purposes of the mDFA was to examine the fractal structure of the potentials recorded from multiple segments. To this end we analyzed the mDFA of the ensemble of potentials recorded from the L4 to L7 segments in the left side, as indicated in insert in Figure 6A. After the left spinal lesion between L5L and L6L, the Hurst value of the mDFA was reduced from 1.178 to 0.969 and remained essentially the same after the lesion in the right side (0.972). 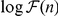 was larger than the values calculated for the single segments (Figures 3, 4, 5), suggesting larger fluctuations or intersegmental synchronizations. We also analyzed the changes produced for the ensemble of 4 sets of potentials obtained from the L5 and L6 segments in both sides (L5L - L5R - L6R - L6L; see insert in Figure 6B). The mDFA Hurst exponents were reduced from 1.173 to 0.946 and 0.917, after the first and second spinal lesions, respectively. Also the fluctuations were significantly reduced for the largest window size (Table 4). Note the mDFA values of the adjacent segments to the lesion (Figure 6B) were reduced more than those located rostrocaudally (Figure 6A).

**Table 4:** 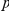
-values for significance tests of fluctuations reduction caused by lesions in L6L and L6R.

mDFA	Left spinal lesion	Right spinal lesion
mDFA L4L-L5L-L6L-L7L	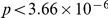	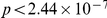
mDFA L5L-L5R-L6R-L6L	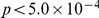	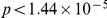

## Discussion

We have shown that detrended fluctuations analysis (DFA) of the spontaneous cord dorsum potentials allows detection of dynamic signatures characteristic of long-range correlations. This may indicate a fractal structure of the neuronal sets involved in the generation of these potentials. As it was shown by means of randomized versions of the experimental data, these long-ranged correlations are not artifacts, but rather a characteristic feature of spontaneous CDPs. As an example of the utility of the method, we analyzed data from one typical experiment aimed to examine the effects of partial spinal lesions. We could show that spinal damage reduced the Hurst exponents of individual spinal segments as well as the correlation length of the signals. 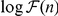, which is an index of the fluctuations of the potentials was also reduced after the spinal lesions. This is in agreement with previous findings, where it was shown that these lesions reduced the correlation between the CDPs recorded from segments adjacent to the spinal lesions [33].

An important feature of this work is the introduction of multichannel detrended fluctuations analysis (mDFA), as a generalization of DFA for multichannel signals. By means of mDFA we studied the effect of long range of correlations in multiple spinal segments measurements of SSA also noticing the presence of a long range correlation structure in intact spinal cord recordings. mDFA also revealed an already envisioned temporal synchronization in the activity of multiple segmental signals, thus revealing spinal communication as a complex coupled phenomenon. To reveal the effect of synchronization in the intact neuroaxis and spinal cord damage we performed mDFA calculations of multiple-segmental signals. These results point out to multiple segments SSA as highly correlated, highly synchronized phenomena for the intact neuroaxis and spinal damage caused both decorrelation and desynchronization of SSA signals.

In contrast to the classical methods, such as correlation and coherence quantification that define a relation between two sets of data, and the classical DFA method that analyzes the correlation dynamics in a single channel, the mDFA method presented here appears to be adequate to examine the synchronization between spontaneous potentials recorded from multiple spinal segments. It thus seems that the mDFA may be useful approach to characterize the functional state of the network under different experimental conditions, such as those induced by acute peripheral nerve or dorsal root section or partial spinal lesions. To test the method we compared mDFA values of multiple-segmental signals before and after spinal lesions in a typical experiment. After the first spinal lesion, the mDFA values were reduced for sets of the segments located rostrally, caudally and also with interposed lesions. 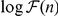, which in this case is an index of the fluctuations of the potentials recorded in the whole ensemble, was also reduced after the first spinal lesion, mainly for sets located rostrally and with interposed lesions. After the second spinal lesion the mDFA values increased for recordings located rostrally or caudally to the lesion, in contrast with the reduced mDFA values for segments adjacent to the lesion. The observed differences in the mDFA for pairs located rostrally and caudally to the lesion can be accounted for by assuming that the rostral segments were subjected to descending influences which may be different from those affecting the segments located caudally to the lesion. The interposed lesions show a reduction in the intersegmental correlation, which was reflected in the reduction in the mDFA 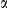 values as well as 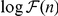, particularly for the largest window sizes. This suggests that the interconnections between the segments adjacent to the spinal lesions were largely reduced. It should be noted that in general the observed changes in mDFA were consistent with the changes in coherence (Figure 3C, 4C and 5C).

The mDFA method has thus revealed as an effective algorithm for the analysis of correlation and synchronization of spontaneous CDPs in different segments. The ensembles involved in the generation of these potentials have a non-random organization of interconnected sets distributed throughout the lumbosacral spinal cord that depends on the overall balance between the descending and segmental inputs, presumably in both sides of the spinal cord [33]. This may explain the inability of a lesion in one side of the spinal cord to completely desynchronize the spontaneous CDPs. The mDFA method may have a potential use to characterize the state of the spinal cord in those instances where a fast quantitative determination of spinal damage is needed. mDFA may have have thus potential use in clinical instances for a fast quantitative determination of spinal damage. More detailed neurophysiological studies are needed to determine the actual mechanisms of spinal synchronization and correlation.
